# Multi-omics profiling reveals epigenetic alterations and a candidate SIRT1-β-catenin axis in bakuchiol ferulate-mediated photoaging protection

**DOI:** 10.3389/fphar.2026.1972459

**Published:** 2026-10-30

**Authors:** Yuting Liang, Yizhen Yan, Zhili Cai, Nan Zhao, Jiangming Zhong, Peng Shu

**Affiliations:** 1 HBN Research Institute and Biological Laboratory, Shenzhen Hujia Technology Co., Ltd., Shenzhen, Guangdong, China; 2 Guangdong Engineering Technology Research Center for Functional Skincare Innovation, Shenzhen Hujia Technology Co., Ltd., Shenzhen, China

**Keywords:** bakuchiol ferulate, epigenetics, methylation, miRNA, photoaging, retinol

## Abstract

Skin photoaging, driven primarily by ultraviolet A (UVA) exposure, involves extracellular matrix degradation, aberrant inflammation, and multilayered epigenetic dysregulation. Bakuchiol ferulate (BF), a novel ester conjugate of bakuchiol (BAK) and a ferulic acid (FA) analog, was synthesized to combine the beneficial properties of BAK and FA. However, its anti-photoaging efficacy and mechanisms of action remain unexplored. Here, we evaluated BF in a UVA-induced nude mouse model using integrated transcriptomic, DNA methylomic, and miRNA profiling, with BAK, FA, and retinol (ROL) included as comparators. BF dose-dependently attenuated UVA-induced photoaging phenotypes and produced greater improvements than BAK or FA across the endpoints examined. Relative to 0.01% ROL, 0.1% BF showed comparable efficacy, whereas 0.4% BF yielded larger improvements in epidermal thickness, dermal collagen content, and inflammatory markers. These phenotypic effects were accompanied by coordinated epigenetic changes, including alterations in miRNA expression and DNA methylation. Integrative network analysis revealed an association between the SIRT1-β-catenin signaling pathway and BF’s anti-photoaging effects. These network-level predictions were in line with molecular observations of β-catenin nuclear accumulation and elevated Cyclin D1 expression, together with reduced miR-34a-5p and miR-450b-3p expression associated with increased SIRT1 and β-catenin protein levels, respectively. Molecular docking further suggested potential binding of BF to SIRT1. Collectively, these findings characterize the multi-layered molecular response to BF in photoaged skin and associate its protective effects with potential engagement of the SIRT1-β-catenin axis. These findings underscore molecular grafting as a rational strategy for engineering phytochemical derivatives with enhanced anti-photoaging profiles, expanding the chemical space of natural product-based skin protective agents.

## 1 Introduction

Skin photoaging, primarily driven by chronic ultraviolet A (UVA) exposure, is characterized by wrinkle formation, extracellular matrix (ECM) degradation, and aberrant inflammation, representing a major dermatological and cosmetic concern. Although retinoids such as retinol (ROL) remain the clinically validated gold-standard anti-photoaging agents (Randhawa et al., 2015), their application is frequently constrained by phototoxicity and skin irritation, which manifest as erythema, desquamation, and stinging sensations. These limitations have sustained the search for safe and effective retinoid alternatives.

Molecular grafting represents an effective strategy to integrate the advantages of distinct molecules and confer novel properties to compounds (Quijas et al., 2022; Jiang et al., 2024). Bakuchiol (BAK) is a plant-derived retinol-like compound (Chaudhuri and Bojanowski, 2014; Dhaliwal et al., 2018), while ferulic acid (FA) is a botanical phenolic acid renowned for its antioxidant capacity (Neopane et al., 2023). Bakuchiol ferulate (BF) is a novel ester synthesized from BAK and an FA analog, designed to couple the complementary bioactivities of both precursors with improved molecular properties. Consistent with this design rationale, our prior characterization demonstrated that BF exhibits enhanced thermal and photostability, a non-phototoxic profile, and no dermal irritation in a 14-day human cumulative patch test even at 1.0% concentration, distinguishing it from ROL which showed phototoxicity and potential irritancy (Zhong et al., 2026). These attributes position BF as a promising and well-tolerated retinoid alternative. However, the molecular mechanisms through which BF exerts its anti-aging effects on skin remain largely unexplored.

Skin photoaging is a complex biological process involving the dysregulation of multi-layered molecular networks. Traditional single-omics approaches often fail to capture the systemic characteristics of this pathology. In contrast, multi-omics integrating transcriptomic, DNA methylation, and miRNA profiling enables systematic dissection of the epigenetic regulatory networks underlying photoaging from multiple layers (Dermitzakis et al., 2025).

Transcriptomic sequencing captures UV-induced gene expression alterations and identifies disrupted signaling pathways, but it cannot distinguish between transient transcriptional responses and long-term epigenetic modifications. DNA methylation, a hallmark epigenetic modification, is critical for maintaining skin homeostasis; chronic UV exposure induces global shifts in the DNA methylome, where methylation changes in promoter regions directly modulate gene transcription (Dermitzakis et al., 2025). Furthermore, miRNAs act as key epigenetic effectors by binding to the 3′-untranslated regions of target mRNAs, thereby negatively regulating gene expression post-transcriptionally. This regulatory mode enables extensive combinatorial control, as individual miRNAs can coordinately downregulate entire functional gene networks, while individual genes may be fine-tuned by multiple miRNAs. Consequently, UV-induced shifts in miRNA expression propagate system-wide transcriptomic perturbations that drive photoaging progression (Gerasymchuk et al., 2020).

Among the molecular regulators implicated in photoaging, SIRT1, an NAD^+^-dependent deacetylase and a well-established longevity regulator, has been recognized as a key regulator of cutaneous responses to UV stress. Repetitive UVA irradiation downregulates SIRT1 expression in human skin and dermal fibroblasts, and UV-driven lipid peroxidation products further impair SIRT1 activity, leading to the accumulation of acetylated proteins and fibroblast senescence (Negre-Salvayre and Salvayre, 2022; Ma et al., 2026). Mechanistically, SIRT1 restrains oxidative stress and cellular senescence by deacetylating transcriptional regulators such as FOXO3a, p53, and PGC-1α, thereby sustaining the expression of antioxidant defense genes (Ma et al., 2026; Lee et al., 2022). In parallel, SIRT1 preserves extracellular matrix homeostasis by suppressing matrix metalloproteinases and limiting collagen degradation, and pharmacological or natural-product-mediated activation of SIRT1 attenuates UV-induced senescence and photodamage in human dermal fibroblasts and mouse skin (Lee et al., 2022; Gilbert et al., 2023).

Canonical Wnt/β-catenin signaling is also implicated in skin aging and photoaging. This pathway sustains fibroblast proliferation and collagen synthesis, whereas UV-induced suppression of Wnt/β-catenin activity promotes fibroblast senescence and ECM deterioration; conversely, restoring pathway activation ameliorates photoaging phenotypes and collagen loss (Liu et al., 2026; Shin et al., 2026). Importantly, β-catenin is an acetylation-sensitive effector, and lysine acetylation enhances its stability, nuclear accumulation, and transcriptional output (You et al., 2022). SIRT1 directly deacetylates β-catenin, and this modification has been shown to govern mesenchymal stem cell fate (Simic et al., 2013), and the SIRT1/β-catenin axis has since been shown to couple sirtuin activity to Wnt signaling output in fibroblasts (Zhang et al., 2024). Collectively, these observations position the SIRT1-β-catenin axis as a potential link between epigenetic dysregulation and the imbalance between cellular senescence and extracellular matrix homeostasis in photoaged skin. Whether this axis is engaged during UVA-induced photoaging, and whether it can be modulated by candidate anti-photoaging agents, remains largely unexplored.

In this study, we established a UVA-induced nude mouse photoaging model to comparatively evaluate whether the molecularly grafted conjugate BF exhibits enhanced anti-photoaging effects relative to BAK and FA, as assessed by skin phenotypic characterization, histopathological examination, and transcriptomic profiling. We further compared BF with ROL across multiple molecular layers, including the transcriptome, miRNAome, and DNA methylome, to delineate similarities and differences in their epigenetic regulatory architectures underlying anti-photoaging activity. Through integrative multi-omics network analysis, we sought to systematically characterize the mechanistic basis of BF-mediated photoaging protection and to examine whether the SIRT1-β-catenin axis is associated with these effects, thereby providing theoretical and experimental foundations for developing novel epigenetic-targeted anti-photoaging ingredients.

## 2 Materials and methods

### 2.1 Animal experiments

BALB/c nude mice (6-weeks-old, male) were acclimatized to the housing environment for 7 days prior to experimentation. UVA irradiation was administered to the dorsal skin using a UV lamp at a frequency of 4 times per week. The UVA intensity was calibrated and expressed as minimal erythema dose (MED), with a progressive escalation protocol adapted from previously established photoaging induction regimens (Xia et al., 2024). Specifically, the protocol comprised weekly escalating doses: 1, 2, 4, and 8 MED (0.45, 0.9, 1.8, and 3.6 J/cm^2^, respectively), culminating in ∼60 MED (27 J/cm^2^). Immediately following each UVA irradiation session, 100 μL of the designated emulsion was applied uniformly to the dorsal skin. On non-irradiation days, the same dosage was administered once daily. The emulsion formulations are provided in Supplementary Table S1.

Molar doses were used for the BF/FA/BAK comparison to enable equimolar, molecular-weight-independent evaluation of intrinsic efficacy, whereas percentage doses were used for the BF/ROL comparison to align with the conventional expression of retinol content in marketed topical products. Molecular weights and dose conversions are provided in Supplementary Table S2. For comparative evaluation of BF, FA, and BAK, mice were randomized into six treatment groups (n = 6): Control, UVA, UVA + FA (5 mM; ∼0.10% w/v), UVA + BAK (5 mM; ∼0.13%), UVA + BF (5 mM; ∼0.22%), and UVA + BF (10 mM; ∼0.43%). For comparative evaluation of BF and ROL, mice were randomized into six treatment groups (n = 6): Control, UVA, UVA + 0.01% ROL, UVA + 0.01% BF, UVA + 0.1% BF, and UVA + 0.4% BF. All treatments were coded and administered by an investigator blinded to treatment allocation, and all Control and UVA groups received vehicle treatment. Cutaneous surface texture of the dorsal skin was quantified using VisioScan VC20 Plus (Germany). The experimental duration was 28 days. No surgical or otherwise painful procedures were performed during the experiment, and anesthesia was therefore not required. At the end of the experiment, all mice were euthanized by gradual-fill CO_2_ inhalation (displacement rate of 20%–30% of the chamber volume per minute), followed by cervical dislocation as a confirmatory physical method, in accordance with AVMA guidelines.

### 2.2 Skin tissue collection and processing

The dorsal skin specimens were harvested with subcutaneous adipose tissue meticulously excised. For histological analysis, skin samples (0.5 × 0.5 cm) were obtained from anatomically consistent locations, flattened between aluminum foil, and fixed in 4% paraformaldehyde at room temperature for 48 h, in preparation for subsequent paraffin embedding and sectioning. Residual skin was rapidly dissected into 0.5 × 0.5 cm fragments, flash-frozen in liquid nitrogen, and archived at −80 °C until use.

### 2.3 Hematoxylin-eosin (H&E) and Masson’s trichrome staining

The fixed skin specimens underwent sequential dehydration in progressively concentrated ethanol, xylene clearing, and paraffin infiltration. Processed tissues were then embedded into paraffin molds and sliced into 5-μm sections. For H&E staining, paraffin sections were first dewaxed in xylene and rehydrated through decreasing ethanol gradients. They were subsequently incubated in hematoxylin (5–10 min), differentiated in 1% hydrochloric acid-ethanol, and counterstained with eosin (1–3 min). For Masson’s trichrome staining, sections were pretreated with potassium dichromate solution (12–18 h), exposed to Weigert’s iron hematoxylin (5 min), and differentiated in 1% hydrochloric acid-ethanol. This was followed by immersion in Ponceau fuchsin (10 min), phosphomolybdic acid differentiation, and aniline blue treatment (30 s). All slides were then dehydrated, rendered transparent with xylene, and coverslipped with neutral balsam for microscopic evaluation. Quantitative assessments of epidermal thickness and collagen deposition were performed using ImageJ software. Experiments, image acquisition and quantification were performed blinded to treatment allocation, with treatment-group assignments revealed only after analysis was complete.

### 2.4 Immunohistochemical (IHC) staining

Deparaffinization, antigen retrieval, and immunohistochemistry were performed using a BOND-III Fully Automated IHC and ISH Staining System (Leica Biosystems, Germany). Endogenous peroxidase activity was quenched with 3% hydrogen peroxide for 10 min. Primary antibodies against p16, p21, IL-1β, and GSK3B (all from Huabio, China) and IL-6 (Abcam, UK) were diluted according to the manufacturer’s instructions and incubated at room temperature for 1 h. Following PBS washes, sections were incubated with biotinylated secondary antibodies (Sino Biological, China) at room temperature for 30 min. Chromogenic detection was performed using a DAB Peroxidase (HRP) Substrate Kit (Beyotime Biotechnology, China), followed by counterstaining with hematoxylin. Quantification of immunostaining was performed using the IHC Toolbox plugin in ImageJ. Experiments, image acquisition and quantification were performed blinded to treatment allocation, with treatment-group assignments revealed only after analysis was complete.

### 2.5 RNA sequencing and analysis

For transcriptomic profiling of BF relative to BAK and FA, RNA-seq was performed on dorsal skin samples from the following groups: Control, UVA, UVA + FA (5 mM), UVA + BAK (5 mM), and UVA + BF (5 mM). For comparative analysis between BF and ROL, RNA-seq was conducted on samples from Control, UVA, UVA + 0.01% ROL, and UVA + 0.4% BF groups. For each group, three biological replicates were randomly selected for library construction and sequencing.

TRIzol reagent (Invitrogen, United States) was used to isolate total RNA, and mRNA fractions were enriched *via* Oligo (dT) magnetic bead capture. cDNA libraries were constructed for PE150 sequencing on the Illumina NovaSeq X Plus platform (Illumina, United States). After quality filtering, sequencing reads were aligned to the mouse GRCm38/mm10 reference genome using HISAT2 (v2.1.0). Gene expression was quantified with StringTie (v2.1.7), and differentially expressed genes were identified using edgeR (v4.4.1), with |log_2_ fold change| > 0.5 and adjusted p < 0.05 considered statistically significant. The resulting gene lists, ranked by log2 fold change, were subsequently subjected to Gene Set Enrichment Analysis (GSEA) to identify pathways significantly enriched in each comparison using clusterProfiler (v4.18.4).

### 2.6 miRNA sequencing and analysis

miRNA sequencing was conducted on dorsal skin samples from the Control, UVA, UVA + 0.01% ROL, and UVA + 0.4% BF groups, using the identical set of mice subjected to RNA-seq analysis. Total RNA was used as input for small RNA library preparation. Adapter ligation was performed at both ends of small RNAs, followed by reverse transcription, PCR amplification, and agarose gel size selection. Cluster generation was performed on the cBot system using the TruSeq PE Cluster Kit v3-cBot-HS (Illumina, United States), followed by SE50 sequencing on the Illumina NovaSeq X Plus platform (Illumina, United States). Raw reads were processed using Cutadapt (v1.18) and fqtrim (v0.9.5) to remove adapter sequences and low-quality bases (quality score < 20), retaining only reads with length >18 nt. Filtered reads were aligned against the Rfam database to annotate and exclude other ncRNAs (rRNA, lincRNA, tRNA, snRNA, snoRNA, and sRNA), and against Repbase to remove repetitive sequences. Clean data were then mapped to the reference genome using miRDeep2 (https://github.com/rajewsky-lab/mirdeep2/tree/master). Differentially expressed miRNAs were identified using edgeR (v4.4.1), with |log_2_ fold change| > 1 and adjusted p < 0.05 considered statistically significant. Putative target genes of these identified miRNAs were retrieved from miRTarBase 2025, a database of experimentally validated miRNA-target interactions (Cui et al., 2025). Functional enrichment analysis of the target genes were performed using clusterProfiler (v4.18.4). Pathways related to Environmental Information Processing were selected for visualization based on the research focus.

### 2.7 Whole-genome bisulfite sequencing (WGBS)

WGBS was conducted on dorsal skin samples from the Control, UVA, UVA + 0.01% ROL, and UVA + 0.4% BF groups, using the identical set of mice subjected to RNA-seq analysis. Genomic DNA was extracted from skin tissue and fragmented into 200–400 bp fragments. Following DNA end repair and adapter ligation, DNA underwent bisulfite conversion, followed by low-cycle PCR amplification. Libraries were then subjected to PE150 sequencing on the Illumina NovaSeq X Plus platform (Illumina, United States).

Raw reads were quality-filtered using Trimmomatic (v0.38) with the following parameters: (i) adapter sequence removal; (ii) exclusion of reads with mean Phred quality score < 15; (iii) 3′ end trimming of bases with quality score < 5; and (iv) removal of reads < 80 bp in length post-trimming. High-quality clean reads were aligned to the reference genome GRCm38/mm10 using BSMAP (v2.90). Differentially methylated regions (DMRs) were identified genome-wide using metilene (v0.2-8), with thresholds of CpG sites ≥ 5, |methylation difference| ≥ 0.1 and p (MWU) < 0.01, where p (MWU) denotes the p-value calculated by the Mann-Whitney U test based on a binary segmentation algorithm (Jühling et al., 2016). Consistent p (MWU)-based filtering has been adopted in previous bisulfite sequencing studies (Buckley et al., 2024; Lopusna et al., 2021). Functional enrichment analysis was performed using clusterProfiler (v4.18.4), and the top 50 significantly enriched pathways were subsequently clustered based on semantic similarity and gene overlap using aPEAR (v1.0) to identify functionally related pathway modules.

### 2.8 Multi-omics integration and network construction

To identify core genes underlying BF-mediated anti-photoaging protection, we integrated transcriptome, miRNAome, and methylome to construct a multi-omics network. Integration was performed using the layer-level significant hits from each omics layer (i.e., differentially expressed genes or miRNAs, and DMRs passing the respective layer-specific thresholds described above). Specifically, for the BF vs. UVA comparison, we selected genes supported by at least two of the following regulatory patterns (and *vice versa*): (1) transcriptomic downregulation, (2) upregulation of miRNAs predicted to target the gene, (3) promoter hypermethylation. Genes were further subjected to protein-protein interaction (PPI) analysis using STRING database (v12.0), where edges with a combined confidence score > 0.4 were retained. The network was further narrowed by intersecting these BF-rescued candidates with genes showing opposite regulatory patterns in the UVA vs. Ctrl comparison (i.e., also supported by ≥ 2 omics layers but with reversed regulatory direction). The resulting intersection genes and their first-order network neighbors genes were retained for final network visualization using Cytoscape (v3.10.4).

### 2.9 Western blot analysis

Skin tissues were homogenized in lysis buffer with PMSF. Total protein was quantified by BCA assay. The denatured protein was separated by SDS-PAGE, and transferred to PVDF membranes. After 5% skim milk blocking, the membranes were incubated with primary antibodies. Immunoreactive bands were visualized using super-sensitive ECL luminescence reagent (Meilunbio, China) and captured with the eBlot system (GenScript, China). Band intensities were quantified using ImageJ.

### 2.10 RT-qPCR of miRNAs

miRNAs were reverse-transcribed using the stem-loop method and the primer sequences are provided in Supplementary Table S3.

### 2.11 Molecular docking and molecular dynamics simulations

The crystal structure of SIRT1 (PDB ID: 4ZZI) was retrieved from the RCSB Protein Data Bank and prepared by removing water molecules and adding hydrogen atoms. Protein and ligand structures were processed in AutoDockTools: Gasteiger-Marsili charges were assigned to the protein, and atom types and charges were calculated for the ligand. The docking grid was centered on the 4TQ binding site (center: 5.604, 52.357, −23.5 Å; size: 40 × 42 × 40 Å; spacing 0.375 Å). Molecular docking was performed using AutoDock Vina (v1.2.7) with an exhaustiveness of 50. Protein-ligand interactions were analyzed with PLIP and visualized in PyMOL. For molecular dynamics simulations, the top BF-SIRT1 complex was solvated in a TIP3P water box with 10 Å padding and neutralized with Na^+^/Cl^−^. Simulations were run in GROMACS (v2023.3) using AMBER14SB for protein and GAFF for BF. Systems were energy-minimized, equilibrated by 100 ps NVT and 100 ps NPT at 310.15 K/1 bar, then simulated for 100 ns. MM/GBSA was calculated on 90–100 ns snapshots, ignoring entropy.

### 2.12 Statistical analysis

Statistical comparisons were carried out in R software (version 4.4.2). Depending on data distribution and variance homogeneity, group comparisons were carried out using one-way ANOVA, Welch’s ANOVA, or the Kruskal–Wallis test, followed by appropriate *post hoc* tests (Tukey’s HSD, Games-Howell, or Dunn’s test, respectively). Results are reported as mean ± standard deviation (SD), with p ≤ 0.05 considered statistically significant and p > 0.05 considered not significant (ns). Asterisk notation was employed to indicate significance levels: * for p < 0.05, ** for p < 0.01, *** for p < 0.001, and **** for p < 0.0001.

## 3 Results

### 3.1 BF attenuates UVA-induced photoaging to a greater extent than BAK and FA *in vivo*

To evaluate the systemic anti-aging potential of BF, we first employed a *C. elegans* lifespan assay, which is a well-established invertebrate model for identifying compounds that modulate evolutionarily conserved aging pathways. Treatment with BF extended the mean lifespan of *C. elegans* by 12.93%, an effect comparable to Resveratrol (Res) (Supplementary Figure S1; Supplementary Table S4), indicating engagement of fundamental longevity programs and prompting us to examine its effects in skin.

To determine whether this systemic bioactivity translates into skin protection, we established a UVA-induced photoaging model in BALB/c nude mice and compared BF with equimolar BAK and FA. UVA radiation induced significant epidermal roughness, desquamation, and wrinkle formation (Figure 1A), all of which were attenuated by BF at 5 mM and 10 mM. Notably, 5 mM BF exhibited minimal desquamation compared with equimolar BAK and FA, while 10 mM BF achieved smooth epidermis without visible desquamation and skin texture comparable to the blank control. Wrinkle severity was assessed using the SEw parameter derived from VisioScan VC20 Plus. BF reduced the SEw change rate relative to baseline (Day 0) in a dose-dependent manner, whereas BAK showed moderate reduction and FA had no detectable effect (Figure 1B). Consistent with these macroscopic findings, H&E and Masson’s trichrome staining showed that BF dose-dependently attenuated UVA-induced epidermal thickening and dermal collagen degradation (Figures 1C–E), and IHC revealed greater reductions in the senescence markers p16 and p21 in BF-treated skin than in the BAK and FA groups (Figures 1F,G). Thus, at equimolar concentrations, BF conferred greater protection against UVA-induced photoaging than BAK and FA, as evidenced by improvements in skin phenotype, tissue histology, and cellular senescence markers.

**FIGURE 1:**
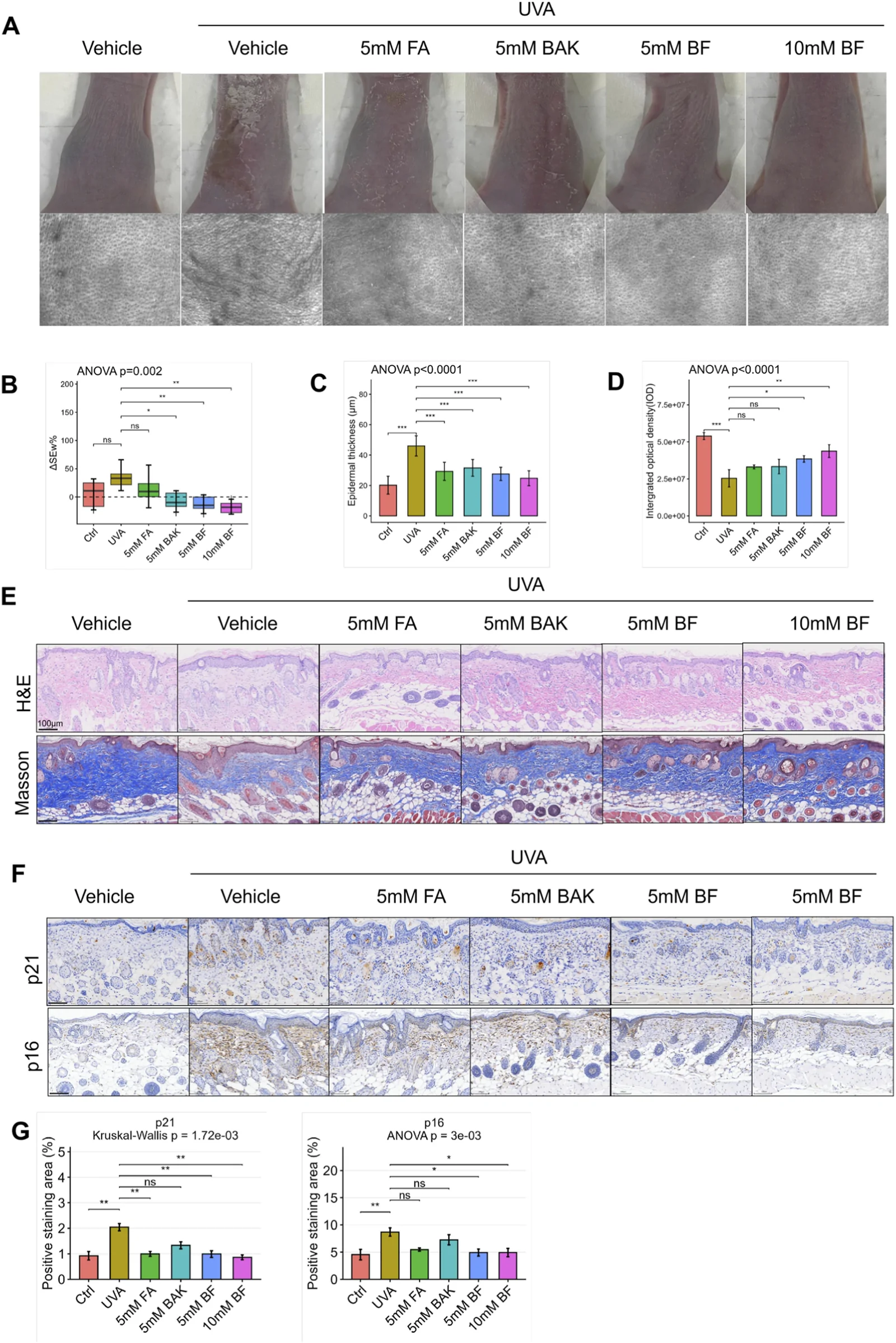
BF ameliorates UVA-induced skin photoaging in a dose-dependent manner, with greater effects than equimolar BAK and FA across phenotypic, histological, and senescence molecular marker analyses. **(A)** Representative gross appearance of nude mouse skin. **(B)** Changes of SEw relative to baseline at day 28. **(C)** Quantification of epidermal thickness in each group. **(D)** Collagen fiber content in skin tissue. **(E)** H&E and Masson’s trichrome staining of skin tissue sections (magnification ×200). **(F)** Representative IHC staining of senescence markers p16 and p21 in dorsal skin (magnification ×200). **(G)** Quantification of p21 and p16 protein expression (Area%) across treatment groups. The data are presented as mean ± SD (n = 6).

### 3.2 BF reverses UVA-induced transcriptomic dysregulation more extensively than BAK and FA

To compare the molecular responses to BF and its parent molecules, we performed transcriptome sequencing. Principal component analysis (PCA) revealed distinct clustering of Control, UVA, and BF groups, whereas the BAK and FA groups separated only partially from the UVA group (Figure 2A), indicating that BF treatment shifted the transcriptomic profile further from the UVA state. We next quantified the reversal of UVA-dysregulated pathways by intersecting significantly enriched GSEA pathways across groups. BF reversed 59.66% of UVA-downregulated pathways, compared with 46.9% for BAK and 28.97% for FA; among UVA-upregulated pathways, the reversal rates were 57.89% for BF, 26.32% for BAK, and 0% for FA (Figures 2B–D). Gene expression heatmaps of the commonly reversed pathways showed a consistent gradient across the three compounds (Figure 2E), in line with the phenotypic differences shown in Figure 1.

**FIGURE 2:**
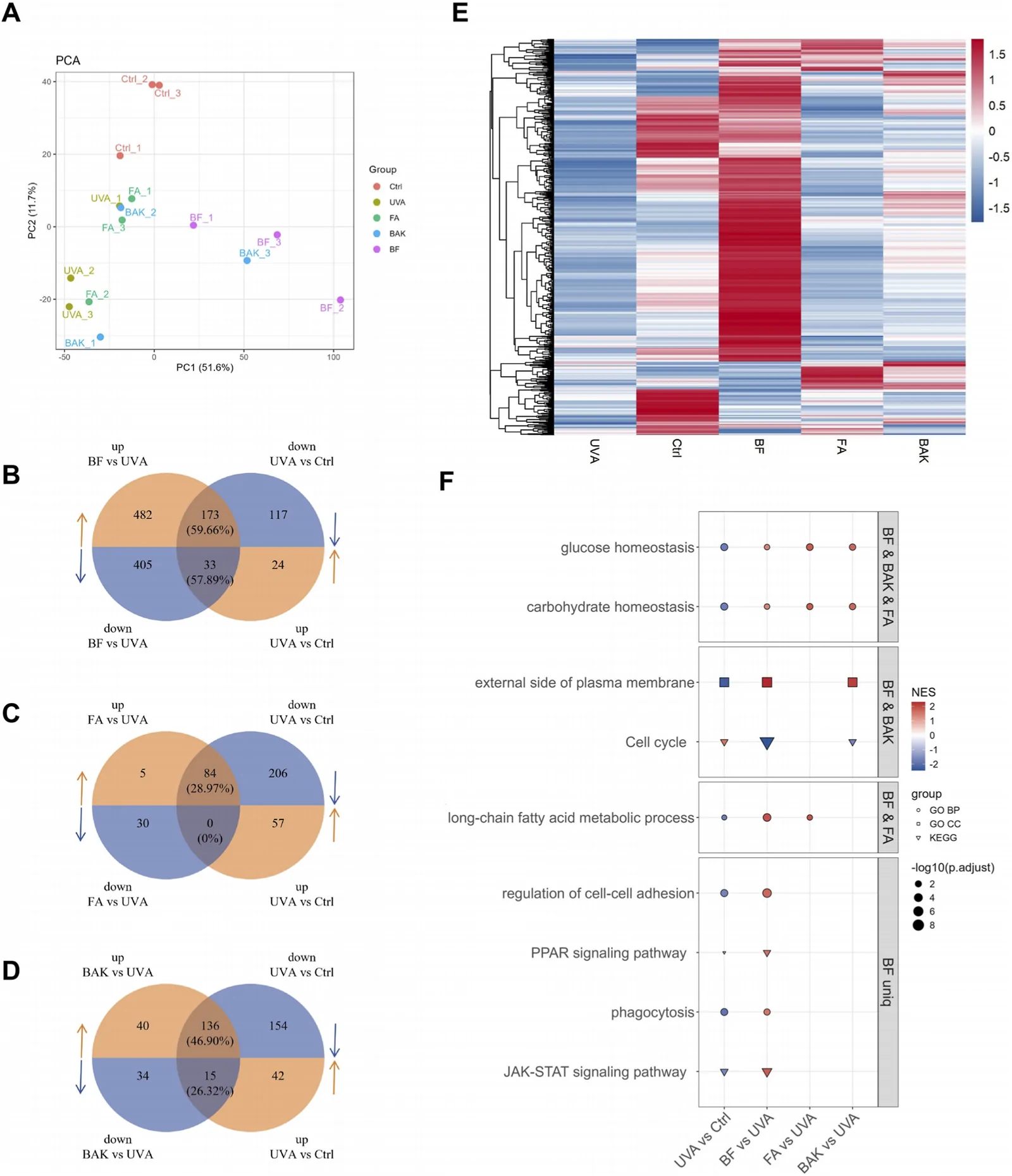
Comparative transcriptomic profiling reveals shared and distinct mechanisms of BF, FA, and BAK against UVA-induced photoaging. **(A)** PCA dimensionality reduction of transcriptomic data. **(B–D)** Rescue of UVA-induced pathway perturbations by BF, FA and BAK, respectively. **(E)** Heatmap showing the expression of genes involved in pathways commonly rescued by BF, BAK, and FA. **(F)** Representative pathways enriched by GSEA. Statistical significance was defined as an adjusted p-value <0.05. Pathways were selected for display based on their established relevance to skin photoaging and extracellular matrix homeostasis.

Representative rescued pathways are shown in Figure 2F. Carbohydrate homeostasis and glucose homeostasis downregulated by UVA were restored by all three compounds, indicating that modulation of cellular energy metabolism is a shared transcriptional response. Both BF and BAK reversed UVA-induced downregulation of the external side of plasma membrane and upregulation of cell cycle pathways, whereas long-chain fatty acid metabolism was shared between BF and FA. In addition, cell adhesion, phagocytosis and JAK-STAT signaling were restored exclusively by BF, indicating that BF engages a broader set of pathways than either parent compound.

### 3.3 BF recapitulates the anti-photoaging effects of ROL and additionally attenuates cutaneous inflammation

Retinoid-induced irritancy is concentration-dependent, with excessive doses eliciting retinoid dermatitis. We therefore first determined an appropriate ROL concentration. As 0.1% ROL is among the concentrations commonly used in clinical treatments, we first evaluated the irritancy of this concentration in mice without UVA exposure. Mild desquamation appeared on Day 7 and became more pronounced by Day 28, with visible erythema (Supplementary Figure S2A), whereas no skin reactions were observed with 0.4% BF. Given that UVA irradiation might aggravate cutaneous irritation, we next evaluated lower ROL concentrations in the UVA-induced photoaging model. Following 14 days of UVA exposure and ROL application, epidermal morphology remained grossly intact in the 0.01% ROL group, whereas 0.03% and 0.05% ROL induced dose-dependent desquamation (Supplementary Figure S2B). Relative to the UVA group, 0.01% ROL attenuated UVA-induced dermal collagen degradation and p16 expression (Supplementary Figure S2B). Based on this combination of tolerability and efficacy, 0.01% ROL was selected for subsequent comparisons.

BF was then tested at three concentrations (0.01%, 0.1%, and 0.4%) alongside 0.01% ROL. UVA irradiation induced pronounced epidermal roughness, desquamation, and wrinkle formation in dorsal skin. BF attenuated these phenotypes in a dose-dependent manner (Figures 3A,B). At 0.01%, BF produced partial improvement, although mild desquamation persisted; at 0.1%, BF yielded gross skin appearance comparable to that of 0.01% ROL; and at 0.4%, BF restored skin appearance to a level comparable to that of the vehicle control (Figures 3A,B). Consistent with these observations, 0.1% BF reduced epidermal thickening and dermal collagen loss to a degree comparable to that of 0.01% ROL, whereas 0.4% BF exerted a greater effect on these parameters (Figures 3C–E). IHC further revealed that 0.01% ROL, as well as 0.1% and 0.4% BF, reduced the senescence markers p16 and p21 (Figures 3F,G). In contrast, the inflammatory cytokines IL-1β and IL-6 were downregulated by 0.1% and 0.4% BF but not by 0.01% ROL (Figures 3F,G). Thus, at the concentrations tested, 0.1% BF paralleled the histological and molecular changes induced by 0.01% ROL, coupled with suppression of dermal inflammatory cytokine expression.

**FIGURE 3:**
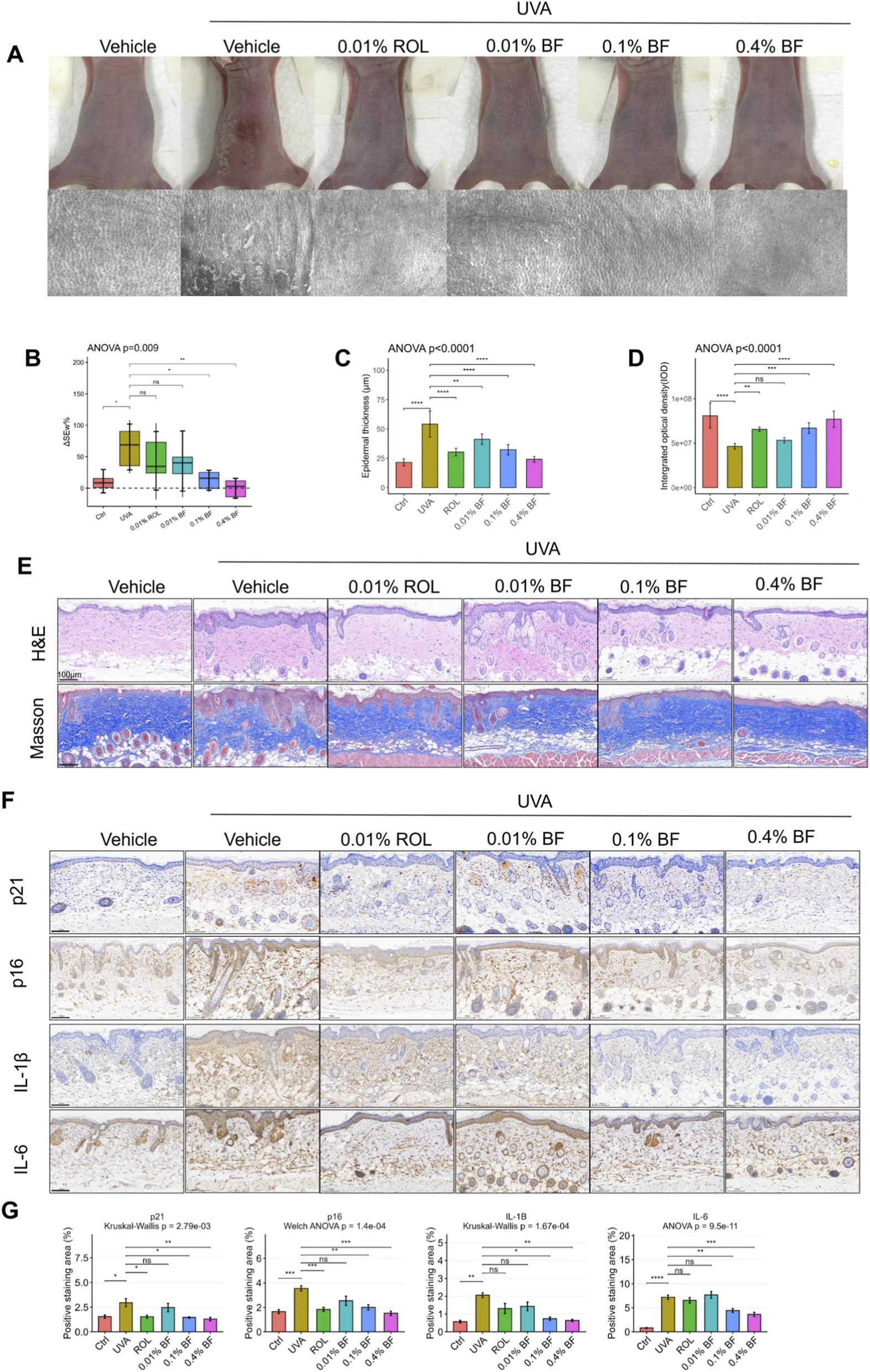
Comparative anti-photoaging efficacy of BF and ROL in UVA-irradiated mouse skin. **(A)** Representative gross appearance of dorsal skin in each treatment group at Day 28. **(B)** Changes in the wrinkle parameter SEw (VisioScan VC20 Plus) relative to baseline (Day 0) at Day 28. **(C,D)** Quantification of epidermal thickness **(C)** and dermal collagen fiber content **(D)** measured from H&E- and Masson’s trichrome-stained sections, respectively. **(E)** Representative images of H&E and Masson’s trichrome staining. **(F)** Representative IHC staining of senescence markers p21, p16, IL-1β and IL-6 in dorsal skin (magnification ×200). **(G)** Quantification of p21, p16, IL-1β and IL-6 protein expression (Area%) across treatment groups. Data are presented as mean ± SD (n = 6).

### 3.4 BF and ROL counteract UVA-induced miRNA dysregulation in association with restored transcriptomic programs

Given that miRNAs represent a key epigenetic regulatory mechanism underlying skin aging (Gerasymchuk et al., 2020; Liang et al., 2024), we performed miRNA sequencing to examine the effects of BF and ROL on miRNA expression in UVA-irradiated skin. PCA revealed distinct clustering across treatment groups, indicating that both UVA exposure and treatment globally altered miRNA expression profiles (Figure 4A). Differential expression analysis identified 43, 41, and 30 miRNAs in UVA vs. Ctrl, BF vs. UVA, and ROL vs. UVA comparisons, respectively. Notably, while UVA irradiation predominantly induced miRNA upregulation, both BF and ROL treatments predominantly led to downregulation (Figure 4B).

**FIGURE 4:**
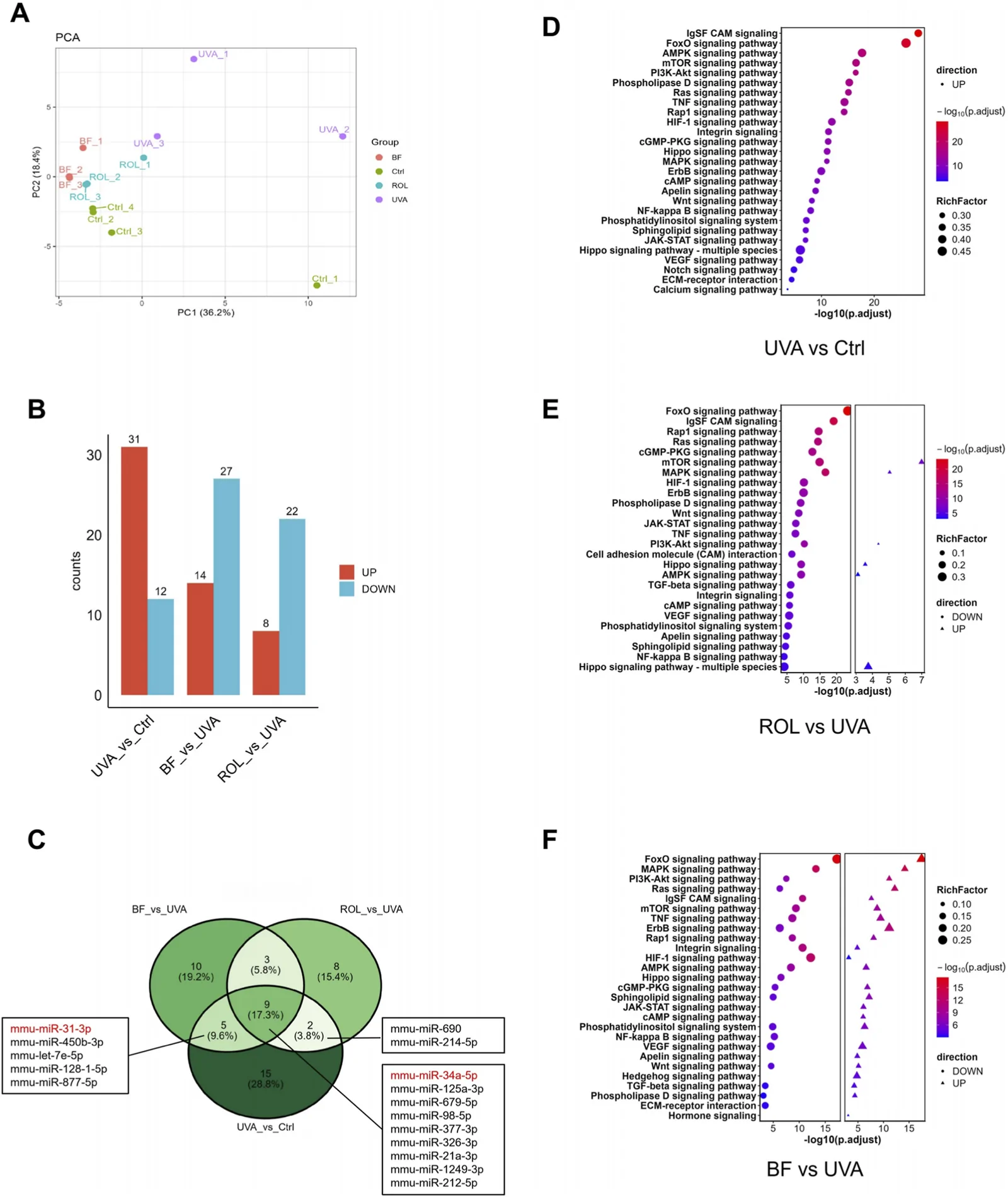
BF and ROL modulate UVA-induced alterations in miRNA expression. **(A)** PCA of miRNA expression profiles. **(B)** Numbers of significantly upregulated and downregulated miRNAs in each pairwise comparison (|log2 fold change| > 1 and adjusted p < 0.05). **(C)** Venn diagrams showing the overlap between upregulated miRNAs in UVA vs. Ctrl and downregulated miRNAs in BF vs. UVA or ROL vs. UVA. **(D–F)** KEGG pathway enrichment analysis of the target genes of differentially expressed miRNAs. Pathways are shown for those classified under the Environmental Information Processing category, with significance defined as adjusted p < 0.05.

To identify miRNAs specifically reversed by the compounds, we intersected the UVA-upregulated miRNAs with those downregulated upon treatment. This intersection yielded nine miRNAs commonly reversed by both BF and ROL (Figure 4C), including the skin aging regulator miR-34a-5p, which modulates cell cycle genes and SIRT1-mediated senescence (Li et al., 2016). Furthermore, five miRNAs were uniquely downregulated by BF. Among these, miR-31-3p is known to be upregulated in aged skin (Liang et al., 2024; Yu et al., 2021), whereas miR-450b-3p and miR-128-5p have been reported as downregulated in aging models (Nidadavolu et al., 2013). In contrast, only two miRNAs were uniquely rescued by ROL, with no known association with skin aging (Figure 4C). These findings indicate that while BF and ROL share a subset of miRNA targets, BF engages a broader spectrum of skin aging-associated miRNAs.

Kyoto Encyclopedia of Genes and Genomes (KEGG) pathway enrichment analysis of the target genes of these miRNAs was performed, focusing on the Environmental Information Processing category. This analysis identified several interconnected signaling pathways, including integrin, FoxO, MAPK, PI3K-Akt, mTOR, and Wnt signaling pathways (Figures 4D–F). These pathways are central to skin homeostasis, spanning integrin-ECM interactions (Quan et al., 2025), PI3K-Akt and mTOR signaling mediated nutrient and growth sensing (Kim and Guan, 2019; Fruman and Rommel, 2014), and Wnt/β-catenin signaling in stem cell maintenance and proliferative responses (Logan and Nusse, 2004; Lim and Nusse, 2013). Concurrent transcriptomic analysis of the same samples further revealed that UVA irradiation downregulated gene programs related to cornified envelope formation and epidermis development, which were conversely upregulated in the BF and ROL groups (Supplementary Figure S3). Collectively, our findings demonstrate that UVA exposure disrupts epidermal homeostasis at both miRNA and transcriptional levels, whereas BF and ROL treatment restores these programs, likely through the modulation of interconnected signaling networks that sustain skin homeostasis.

### 3.5 BF partially restores UVA-induced DNA methylation alterations in skin

To investigate the methylation-level responses to BF and ROL, we performed WGBS on skin samples. Global CpG island (CGI) methylation analysis showed that UVA irradiation reduced CGI methylation levels, consistent with previous reports (Vandiver et al., 2015), whereas the BF and ROL groups exhibited intermediate methylation levels between the UVA and control groups (Figure 5A), suggesting partial reversal of UVA-induced methylation changes.

**FIGURE 5:**
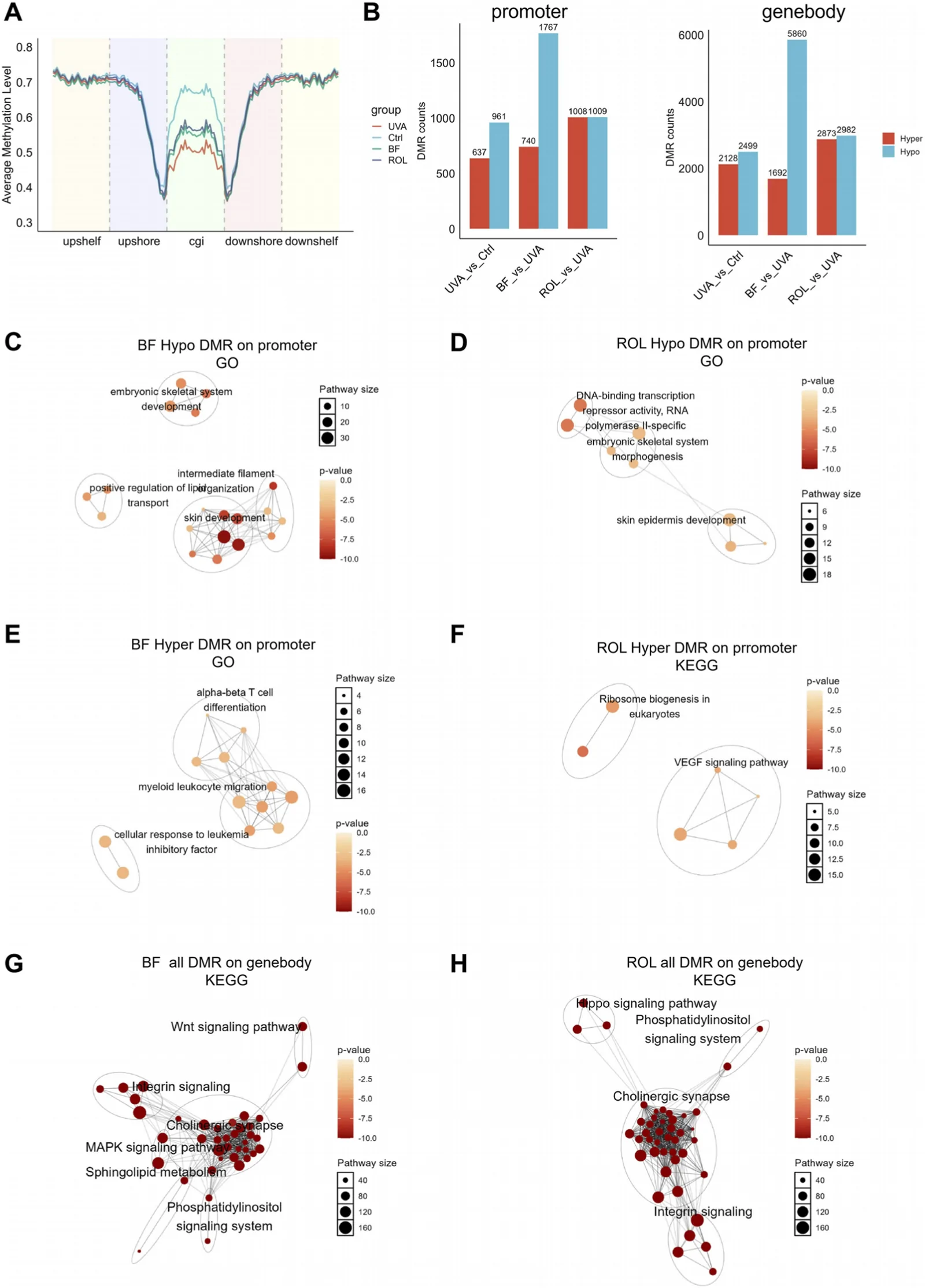
The DNA methylation patterns altered by BF and ROL treatment. **(A)** Genome-wide distribution of CGI methylation levels across treatment groups. **(B)** Number of DMRs annotated to promoter and gene body regions. **(C–H)** Network clustering of GO enrichment analysis for DMRs annotated to promoter **(C–F)** and gene body **(G,H)** regions, generated using aPEAR. Each node represents a significantly enriched pathway; edge width reflects the number of overlapping genes between pathways. Only the top 50 pathways (ranked by adjusted p-value) are shown for cluster visualization, with the lowest-p pathway used as the cluster label.

Differential methylation analysis identified the largest number of DMRs in the BF vs. UVA comparison (Figure 5B), suggesting that BF treatment was accompanied by widespread methylation changes. Functional enrichment analysis of genes with altered promoter methylation revealed distinct patterns between BF and ROL. In both treatment groups, promoter-hypomethylated genes, which are generally associated with transcriptional activation, were enriched for biological processes related to epidermis development (Figures 5C,D). This pattern parallels the transcriptomic upregulation of epidermis development pathways (Supplementary Figure S3) and suggests that promoter demethylation of epidermal development genes may contribute to the restoration of epidermal homeostasis in both treatment groups. By contrast, promoter-hypermethylated genes in the BF group were specifically enriched for pathways including myeloid leukocyte migration and cellular response to leukemia inhibitory factor pathway (Figure 5E), suggesting that BF may attenuate inflammatory responses through promoter methylation-mediated transcriptional repression. No comparable enrichment was observed in the ROL group (Figure 5F), indicating a differential methylation-associated anti-inflammatory pattern between the two compounds, consistent with the immunohistochemical reduction of IL-1β and IL-6 observed in the BF but not the ROL group (Figures 3F,G).

Given that the relationship between gene body methylation and transcription is context-dependent and does not follow a simple directional pattern (Dermitzakis et al., 2025; Jones, 2012; Lev Maor et al., 2015), enrichment analysis was performed on all genes with altered gene body methylation without stratification by methylation direction. Enrichment analysis revealed BF treatment group enrichment in integrin signaling, Wnt signaling, sphingolipid metabolism, and phosphatidylinositol signaling system pathways (Figure 5G), whereas the ROL group showed enrichment in integrin and phosphatidylinositol signaling systems (Figure 5H). The overlap of integrin and Wnt signaling between methylation-associated genes and miRNA targets points to these pathways as potential nodes of multi-layered epigenetic regulation.

The WGBS data indicate that BF treatment is associated with widespread methylation changes affecting epidermal development, inflammation-related, and integrin/Wnt signaling genes, with patterns that partially overlap the transcriptional and miRNA-level alterations described above.

### 3.6 Integrated multi-omics and molecular validation suggest an association between the SIRT1-β-catenin axis and BF’s anti-photoaging effects

To identify candidate regulators underlying BF-mediated photoaging protection, we constructed an integrated miRNA-transcriptome-methylome regulatory network. Genes supported by evidence from at least two omics layers in the BF vs. UVA comparison were retained and intersected with genes showing opposite regulatory patterns in the Ctrl vs. UVA comparison, yielding six genes with reversed regulatory patterns, which together with their first-order neighbors were subjected to network analysis (Figure 6A). Notably, PPI analysis pointed to an association between SIRT1 and β-catenin (Figure 6A), and this association was retained at a high-confidence threshold (combined score > 0.7; combined score = 0.969; Supplementary Figure S4). Meanwhile, β-catenin (Ctnnb1) and its negative regulator GSK3B exhibited the highest degree centrality in the network (Figure 6A).

**FIGURE 6:**
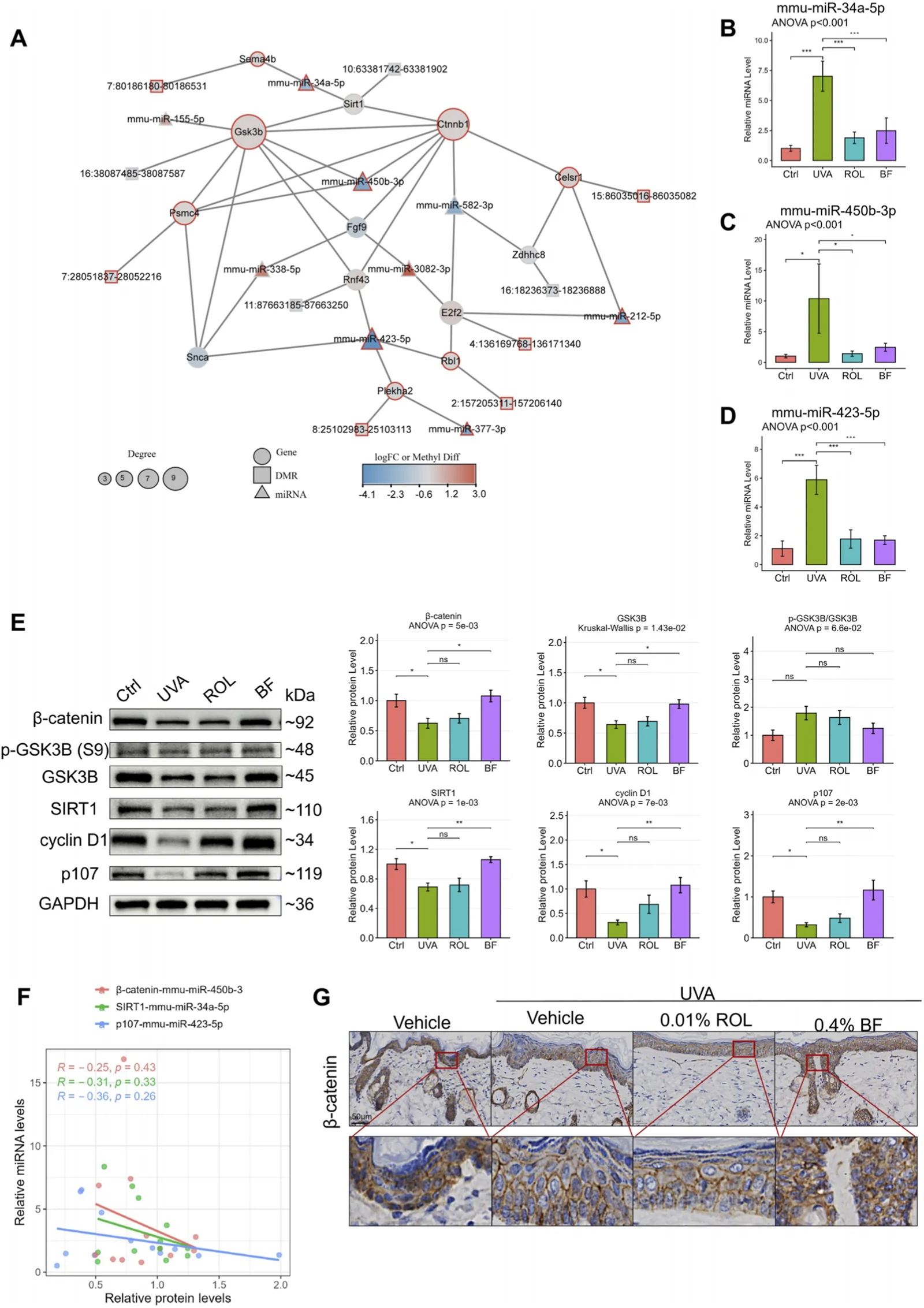
Integrated multi-omics network analysis and molecular assessment of the SIRT1-β-catenin axis. **(A)** Integrated miRNA-transcriptome-methylome regulatory network. Color bars indicate log2 fold change (transcriptome and miRNA) or differential methylation rate (WGBS) in the BF vs. UVA comparison. Red borders mark genes reversed in the UVA vs. Ctrl comparison; node size reflects degree centrality. **(B–D)** RT-qPCR quantification of mmu-miR-34a-5p **(B)**, mmu-miR-423-5p **(C)**, and mmu-miR-450b-3p **(D)**, normalized to U6 and expressed relative to the Ctrl group (n = 3). **(E)** Western blot analysis and quantification of SIRT1, β-catenin, total GSK3B, phospho-GSK3B (Ser9), and Cyclin D1 (n = 6); GAPDH was used as the loading control, and phospho-GSK3B was normalized to total GSK3B. **(F)** Correlation between miRNA-protein expression levels. Pearson correlation analysis was performed to evaluate the relationship between miRNA and protein expression levels across individual samples. Negative correlation trends were observed for all miRNA-target pairs, consistent with miRNA-mediated repression. **(G)** Representative IHC staining of β-catenin in dorsal skin sections (magnification ×400). The BF group appeared to show increased nuclear-associated β-catenin staining compared with the UVA group, whereas the ROL group showed a relatively more membrane-associated staining pattern.

miRNA-mediated de-repression appeared to contribute to the activation of this axis. The network predicted that mmu-miR-34a-5p targets Sirt1, mmu-miR-450b-3p targets both Gsk3b and Ctnnb1, and mmu-miR-423-5p targets Rbl1 (Figure 6A). For each predicted miRNA-target pair, reduced miRNA levels paralleled increased abundance of the corresponding target protein, consistent with relief of miRNA-mediated repression (Figures 6B–F). Western blot analysis revealed that both SIRT1 and β-catenin were concomitantly upregulated in BF-treated skin (Figure 6E). Immunohistochemical staining further indicated increased nuclear localization of β-catenin (Figure 6G), and the downstream transcriptional target Cyclin D1 (CCND1) was likewise elevated, consistent with enhanced β-catenin transcriptional activity in BF-treated skin (Figure 6E).

To dissect the mechanism of β-catenin upregulation, we examined GSK3B, a core component of the β-catenin destruction complex. Total GSK3B protein was increased in the BF group; however, inhibitory phosphorylation of GSK3B at Ser9 did not differ significantly from the UVA group (Figure 6E). This suggests that Ser9-mediated inhibition of GSK3B does not fully account for the β-catenin accumulation observed in BF-treated skin.

### 3.7 BF is predicted to bind to the canonical site for SIRT1-activating compounds (STACs)

Re-docking of 4TQ reproduced the crystallographic pose with an RMSD of 0.25 Å (Supplementary Figure S5), supporting the reliability of the docking protocol. Molecular docking showed that BF bound SIRT1 with a predicted docking score of −9.16 kcal/mol, comparable to but weaker than that of the co-crystallized STAC 4TQ (−11.32 kcal/mol). Interaction analysis identified contacts with Leu206, Leu215, Ile223, Ile227, and Glu230 (Figure 7A), residues that define the canonical STAC-binding site (Dai et al., 2015). During the 100-ns MD simulation, the BF-SIRT1 complex remained stable, and BF persisted in the same hydrophobic pocket mainly through van der Waals/hydrophobic contacts, with the key STAC-site interactions maintained throughout the trajectory (Figures 7B–G).

**FIGURE 7:**
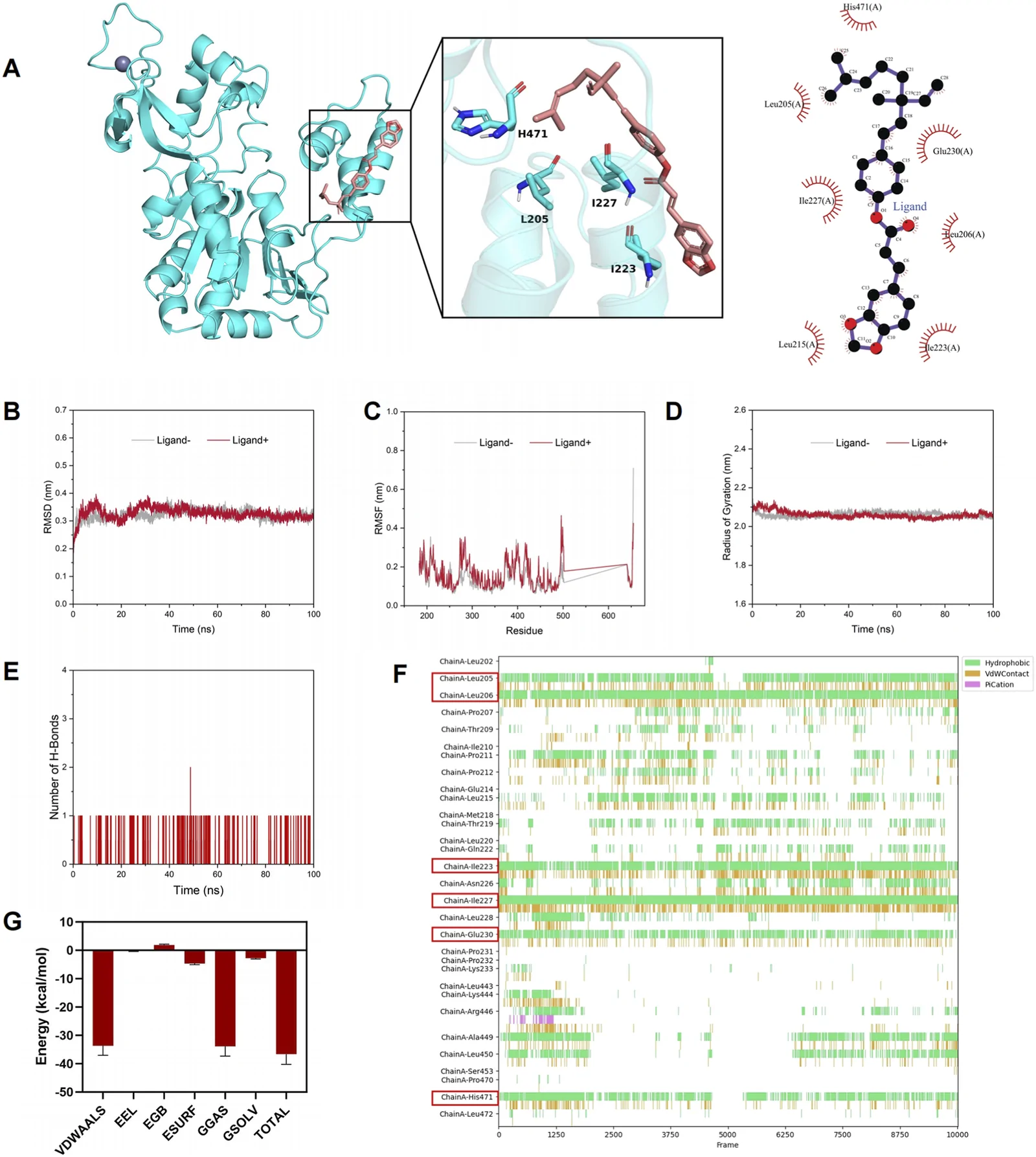
Molecular docking and MD simulation analysis of BF binding to SIRT1 (PDB 4ZZI). **(A)** Three-dimensional binding pose of the BF-SIRT1 complex and PLIP interaction analysis. **(B)** RMSD curves during the 100-ns simulation; after entering the stable fluctuation stage, the RMSD remained within 0.3–0.35 nm. **(C)** Per-residue RMSF analysis. Except for the C-terminal region (∼residue 650) and local regions surrounding the binding pocket, RMSF values were generally low. **(D)** Radius of gyration (Rg) analysis. The Rg curves with and without BF were highly overlapped with small fluctuations. **(E)** Number of hydrogen bonds between BF and SIRT1. **(F)** Interaction fingerprint analysis showing that BF maintained hydrophobic/van der Waals contacts with Leu205, Leu206, Ile223, Ile227, Glu230, and His471 throughout most of the trajectory. **(G)** MM/GBSA binding free energy analysis using the 90–100 ns trajectory, indicating that binding was dominated by van der Waals interactions.

## 4 Discussion

UVA exposure induces premature skin aging characterized by epidermal thickening, dermal collagen degradation, and inflammatory dysregulation. Although retinoids remain established anti-photoaging agents (Lin et al., 2025), their use is constrained by concentration-dependent cutaneous irritation (Zasada et al., 2020). Plant-derived compounds, including BAK and FA, have been explored as gentler alternatives (Dhaliwal et al., 2018; Brown et al., 2023; Zduńska et al., 2018; Zduńska-Pęciak et al., 2022). In this study, we evaluated BF, a grafted conjugate of BAK and an FA analog, in a UVA-induced mouse photoaging model. We employed BALB/c nude mice, which are widely used in skin photoaging research, to avoid depilation-induced skin irritation that could confound topical treatment outcomes. Their keratinocyte-mediated innate immune responses remain intact, permitting reliable assessment of inflammatory cytokines induced by UVA exposure (Liu et al., 2024). BF attenuated photoaging phenotypes more effectively than equimolar concentrations of its parent molecules and, at the concentrations tested, reproduced the effects of 0.01% ROL while additionally reducing dermal inflammatory cytokines. Multi-omics profiling further associates these protective effects with coordinated miRNA, DNA methylation, and transcriptomic changes related to skin homeostasis programs and the SIRT1-β-catenin axis. This work addresses the challenge of uncoupling anti-aging efficacy from cutaneous irritation, demonstrating that molecular hybridization represents an effective strategy for integrating the advantages of distinct bioactive compounds to develop anti-photoaging agents.

The hybrid architecture of BF raises the question of whether covalent integration of BAK and FA analogs confers a target profile distinct from the parent compounds. Molecular hybridization has been reported to yield entities whose activities are not merely additive but qualitatively distinct from those of the parent molecules, including altered selectivity and emergent modes of action (Tietze et al., 2003; Meunier, 2007; Mishra and Singh, 2016). In addition, structural modification of ferulic acid itself has produced derivatives with bioactivity profiles exceeding those of the parent compound (Shikama et al., 2025). Consistent with this precedent, BF outperformed equimolar BAK and FA across gross morphology, histology, senescence markers, and comparative transcriptomics, and produced extensive reversal of UVA-dysregulated pathways. These observations are consistent with the possibility that molecular hybridization expands the biological repertoire of plant-derived bioactives. However, our study design did not include a physical-mixture control in which equimolar BAK and FA are co-administered in unlinked form, and we therefore cannot exclude that simultaneous delivery of the two parent molecules would recapitulate part or all of the effects observed with BF.

In addition to biological activity, skin tolerability is an important consideration for the practical application of topical anti-aging agents. In a 14-day human patch test on the volar forearm, all tested BF concentrations (0.05%–1%) were classified as non-irritating, whereas ROL showed a concentration-dependent increase in irritancy, reaching a “slightly irritating” classification at 0.3% (Zhong et al., 2026). In this study, 28-day treatment with 0.4% BF on mouse dorsal skin produced no adverse reactions, whereas both 0.05% and 0.03% ROL caused adverse reactions, prompting us to use 0.01% ROL as the final comparator dose. This dose selection should be interpreted in the context of species differences between mouse and human skin. Mouse skin has a thinner stratum corneum and epidermis than human skin (9 vs. 17 μm and 29 vs. 47 μm, respectively), resulting in higher permeability (Godin and Touitou, 2007). Moreover, the proportion of the dorsal surface area exposed to the formulation in mice is substantially larger relative to their total body surface area than in humans, further amplifying the risk of retinol-induced irritation. These species-specific factors limit the generalizability of our dose comparison to real-world retinol usage. However, vehicle-controlled clinical studies have demonstrated significant improvement of photoaged skin with retinol concentrations as low as 0.075%–0.1% (Kikuchi et al., 2009; Farris et al., 2024), which is within roughly one order of magnitude of the 0.01% ROL used here. Therefore, whether BF matches the clinical efficacy of ROL remains to be determined in comparative clinical studies.

Mechanistically, BF and ROL diverged in their effects on inflammatory responses, with IL-1β and IL-6 reduced by BF but not by ROL, likely reflecting their distinct pharmacological mechanisms. ROL exerts its effects *via* metabolic conversion to retinoic acid and subsequent RAR activation, which mediates anti-aging benefits while concomitantly inducing potential pro-inflammatory signaling (Babina et al., 2015). By contrast, BF exhibited promoter hypermethylation of inflammation-related genes, a pattern not detected in the ROL group, raising the possibility of an epigenetically mediated anti-inflammatory component. This interpretation aligns with evidence linking promoter DNA methylation to inflammation resolution (Pfalzer et al., 2014); however, the methylation changes reported here are correlative, and their causal role in cytokine suppression requires further investigation. In parallel, promoter hypomethylation of epidermal development genes in both treatment groups paralleled their transcriptomic upregulation, and the overlap of integrin and Wnt signaling between methylation-associated genes and miRNA targets identifies these pathways as nodes of multi-layered epigenetic regulation.

Integrated network analysis implicated Wnt/β-catenin signaling as a candidate pathway in BF photoaging treatment. This pathway governs cell proliferation, differentiation, and stem cell self-renewal, and its output is attenuated in aged and photoaged skin, with diminished signaling closely linked to fibroblast senescence and cell cycle arrest (Shin et al., 2026; Zhong et al., 2025; You et al., 2025). miRNAs are known to modulate this pathway by targeting its core components (Wang et al., 2021; Mahmood et al., 2015; Cha et al., 2012). In BF-treated skin, downregulation of miR-450b-3p, predicted to target Ctnnb1 and Gsk3b, paralleled increased β-catenin protein abundance, while reduced miR-34a-5p paralleled increased SIRT1. These reciprocal expression patterns are consistent with relief of miRNA-mediated repression across the SIRT1-β-catenin network, although the direct regulatory relationships remain to be established. SIRT1-mediated deacetylation of β-catenin can either promote or suppress its nuclear accumulation in a context-dependent manner (You et al., 2022; Simic et al., 2013). In the present UVA model, we observed increased nuclear β-catenin in BF-treated skin, though whether this reflects SIRT1-mediated deacetylation requires further investigation. Upon nuclear entry, β-catenin complexes with TCF/LEF transcription factors, converting the TCF repressor complex into an activator that drives target genes including Cyclin D1 (Tetsu and McCormick, 1999). Consistent with this transcriptional mechanism, we detected elevated Cyclin D1 protein levels in BF-treated skin, further supporting β-catenin activation. These findings align with previous reports showing that Wnt/β-catenin activation increases β-catenin, cyclin D1, and c-myc expression in skin (Zhang et al., 2011), thereby supporting cell proliferation and the restoration of skin homeostasis (Xu et al., 2014; Pulawska-Czub et al., 2026).

Notably, unchanged GSK3B Ser9 phosphorylation is not necessarily incompatible with β-catenin accumulation. The Axin scaffold has been shown to allosterically protect destruction-complex-associated GSK3B from inhibitory Ser9 phosphorylation, so pGSK3B (Ser9) levels do not necessarily reflect the activity of the GSK3B pool that phosphorylates β-catenin (Gavagan et al., 2023). We therefore favor the interpretation that β-catenin accumulation reflects inhibition of β-catenin degradation rather than Ser9-mediated changes in GSK3B activity, although the precise mechanism in BF-treated skin remains unresolved. Two speculative models are presented as untested hypotheses in Figure 8. One is Wnt-dependent disassembly of the destruction complex (Stamos and Weis, 2013). The other is GSK3B-mediated suppression of GID complex-dependent β-catenin degradation, in which GSK3B binding to the GID E3 ligases MAEA and RMND5A antagonizes β-catenin recognition (Shimizu and Shibuya, 2025). Future studies employing co-immunoprecipitation of Axin-GSK3B or GSK3B-GID components from skin lysates will be necessary to test these hypotheses.

**FIGURE 8:**
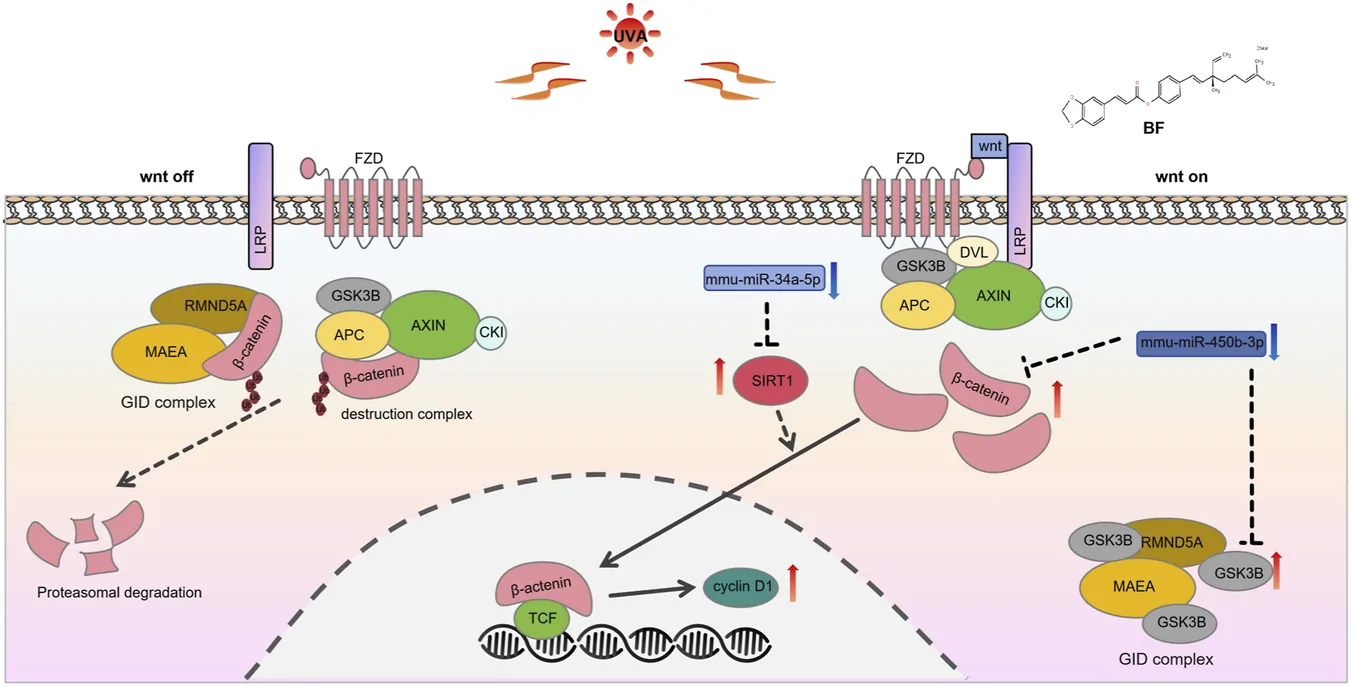
Proposed working model of the SIRT1-β-catenin axis in BF-mediated photoaging protection. The model integrates multi-omics findings from BF-treated skin, highlighting miRNA-SIRT1-β-catenin regulatory connections, β-catenin nuclear translocation and Cyclin D1 activation. Two hypothetical mechanisms for β-catenin stabilization under elevated GSK3B are shown. Dashed arrows denote speculative mechanisms not experimentally validated in this study.

Several limitations of this study warrant consideration. The absence of an equimolar physical mixture of the parent compounds indicates that the enhanced efficacy of BF cannot be definitively attributed to covalent conjugation. To determine whether covalent conjugation confers distinct skin pharmacokinetics compared with the parent compounds, a comparison incorporating permeation assessment in 3D skin models and Raman spectroscopic imaging will be required. Whether these pharmacokinetic differences ultimately translate into improved efficacy will need to be addressed in separate efficacy studies. The multi-omics analyses were performed with a relatively small sample size (n = 3 per group) without *a priori* power analysis, which may increase the risk of false discoveries. The SIRT1-β-catenin axis was inferred from integrative computational and expression analyses, including molecular docking suggesting that BF acts as a potential SIRT1 activator, and pharmacological or loss-of-function experiments will be necessary to establish a causal role for this axis in the effects of BF. Finally, whether BF matches the clinical efficacy of ROL remains to be determined in comparative clinical studies, as discussed above.

## 5 Conclusion

In this study, we evaluated BF in a UVA-induced photoaging model in nude mice and demonstrated that BF produced greater improvements in photoaging phenotypes than equimolar BAK and FA, and achieved anti-photoaging efficacy comparable to that of 0.01% ROL in our model. Integrated multi-omics profiling revealed that these protective effects are associated with coordinated epigenetic alterations. Molecular-level expression analyses and molecular docking were consistent with an association between the SIRT1-β-catenin axis and BF’s anti-photoaging activity. Collectively, these findings provide a multi-layered molecular characterization of BF in skin photoaging and establish a foundation for future mechanistic interrogation of the identified epigenetic and signaling networks.

## Data Availability

The multi-omics datasets generated in this study, including transcriptome, miRNA, and DNA methylome profiles of mouse skins have been deposited in the Sequence Read Archive (SRA) under BioProject PRJNA1474112.
