# Supplementary material for: Multi-omics profiling reveals epigenetic alterations and a candidate SIRT1-β-catenin axis in bakuchiol ferulate-mediated photoaging protection
Source: Front Pharmacol. 2026 Oct 30;17:1972459. doi: 10.3389/fphar.2026.1972459 (PMC13759829; doi:10.3389/fphar.2026.1972459)
Supplement: Supplementary file 1 [file Supplementaryfile1.docx]

**Supplementary Methods**

**Lifespan Assay of *C. elegans***

N2 wild-type *C. elegans* were cultured at 20°C until the L4 larval stage, then transferred to nematode growth medium plates containing the respective drug treatments. The plates were supplemented with 5-fluoro-2-deoxyuridine (FUDR, Aladdin, China) to prevent egg laying. BF stock solutions were prepared in 0.2% DMSO and mixed with OP50 bacterial suspension at a 1:3 ratio (v/v) immediately before use, yielding a final DMSO concentration of 0.05% across all groups. The blank control group received OP50 supplemented with 0.05% DMSO, and 100 μM resveratrol served as the positive control. Each plate received 100 μL of the prepared OP50 mixture.

The worms were scored as dead when they failed to respond to gentle touch with a platinum wire pick. The number of deaths was recorded each day. Survival curves were estimated using the Kaplan-Meier method, and differences between groups were assessed by the Log-rank (Mantel-Cox) test. All statistical analyses were performed using R software (version 4.4.2) with the survminer package (0.5.2). A p value < 0.05 was considered statistically significant.

**Preparation of the Emulsion Formulations**

The phase A, B, and C ingredients were weighed into separate containers according to Supplementary Table 1. Phase A was heated to 75°C with stirring and held for 15 min to ensure uniform dispersion and the absence of crystalline particles. Phase B was heated to 75°C until completely dissolved and homogeneous. At 75°C, phase B was added to phase A under low-speed homogenization, followed by high-speed homogenization for 5 min with continuous stirring. The mixture was cooled to 45°C under moderate stirring, and phase C was added (pre-dissolved at room temperature to clarity, where applicable). Cooling was continued to 30°C, after which the emulsion was degassed under vacuum to remove entrapped air and immediately filled and sealed. Throughout the procedure, light exposure was minimized and heating of phase B was kept brief to prevent degradation of the active ingredients.

**Supplementary** **Figures and Tables**

**Supplementary** **Figure 1.** The effect of BF on the lifespan of *C. elegans*.

**Supplementary Figure 2**. Determination of the optimal ROL concentration for anti-photoaging efficacy with minimal skin irritation. (A). Comparison of skin irritation between 0.4% BF and 0.1% ROL following 28 days of treatment without UVA irradiation. (B). Dose-dependent assessment of retinol-induced cutaneous irritation and protective effects against photoaging following 14 days of combined UVA exposure and treatment in mice.

**Supplementary Figure 3**. GSEA of transcriptome data reveals that BF and ROL reversed UVA-induced downregulation of cornified envelope formation pathways (A-C) and epidermis development (D-F).

**Supplementary Figure 4**. STRING-based PPI network analysis (combine score > 0.7). The Sirt1-Ctnnb1 (β-catenin) interaction was retained at this stringent cutoff (combined score = 0.969), supporting the robustness of their connectivity.

**Supplementary Figure 5**. Validation of the SIRT1 molecular docking protocol. The co-crystallized SIRT1 activator 4TQ from PDB 4ZZI was re-docked into its native binding site.

**Supplementary Table 1 The emulsion formulations for each experimental group (per 100 mL).**

| Phase | Ingredient | 0.01% ROL | 5mM FA | 5mM BAK | 5mM BF | Vehicle |
| --- | --- | --- | --- | --- | --- | --- |
| A | deionized water | 81.7 | 81.5 | 81.6 | 81.5 | 81.7 |
| A | POLYACRYLATE CROSSPOLYMER-6 | 0.2 | 0.2 | 0.2 | 0.2 | 0.2 |
| A | HYDROXYACETOPHENONE | 0.5 | 0.5 | 0.5 | 0.5 | 0.5 |
| A | 1,2-HEXANEDIOL | 0.5 | 0.5 | 0.5 | 0.5 | 0.5 |
| A | BUTYLENE GLYCOL | 8 | 8 | 8 | 8 | 8 |
| A | PELLICER™ LB-10 | 0.1 | 0.1 | 0.1 | 0.1 | 0.1 |
| A | FERULIC ACID | \ | 0.1 | \ | \ | \ |
| B | CAPRYLIC/CAPRIC TRIGLYCERIDE | 7 | 7 | 7 | 7 | 7 |
| B | Olivem 1000 | 1.5 | 1.5 | 1.5 | 1.5 | 1.5 |
| B | RETINOL | 0.01 | \ | \ | \ | \ |
| B | BAKUCHIOL | \ | \ | 0.13 | \ | \ |
| B | BAKUCHIOL FERULATE | \ | \ | \ | 0.22 | \ |
| C | CITRIC ACID | \ | 0.01 | \ | \ | \ |
| C | ARGININE | \ | 0.09 | \ | \ | \ |
|  | Total weight (g) | 99.5 | 99.5 | 99.5 | 99.5 | 99.5 |
|  | Total volume (mL) | 100 | 100 | 100 | 100 | 100 |

*Values are expressed in grams (g). The density of the emulsion is approximately ρ ≈ 0.995 g/mL. Therefore, a total weight of 99.5 g corresponds to a final volume of approximately 100 mL. The 5 mM BF formulation is shown as a representative example; all other concentrations were prepared by adjusting the mass of the active ingredient per 100 mL according to the conversions in Supplementary Table 2, with all other components unchanged and deionized water adjusted to maintain a total weight of 99.5 g.*

**Supplementary Table 2 Concentration conversion across experimental comparisons**

| Chemical | Molecular Weight (g/mol) | Concentration (mM) | Concentration (% w/v) |
| --- | --- | --- | --- |
| BF | 430.21 | **5** | 0.22% |
| BF | 430.21 | **10** | 0.43% |
| FA | 194.18 | **5** | 0.10% |
| BAK | 256.38 | **5** | 0.13% |
| BF | 430.21 | 0.23 | **0.01%** |
| BF | 430.21 | 2.32 | **0.10%** |
| BF | 430.21 | 9.30 | **0.40%** |
| ROL | 286.45 | 0.35 | **0.01%** |

**Supplementary Table 3** Primer sequences used for qPCR analysis.

| miRNAs | Forward primer (5′→3′) | Reverse primer (5′→3′) |
| --- | --- | --- |
| U6 | CTCGCTTCGGCAGCACA | AACGCTTCACGAATTTGCGT |
| mmu-miR-34a-5p | ACACTCCAGCTGGGTGGCAGTGTCTTAGCT | TGGTGTCGTGGAGTCG |
| mmu-miR-450b-3p | ACACTCCAGCTGGGATTGGGAACATTTTGC | TGGTGTCGTGGAGTCG |
| mmu-miR-423-5p | ACACTCCAGCTGGGTGAGGGGCAGAGAGCGA | TGGTGTCGTGGAGTCG |

**Supplementary Table 4** Effects of BF on the lifespan of *C. elegans*

| **Group** | **N** | **Mean ± SE** | **Rate(%)** | ***p* value (log-rank significance)** |
| --- | --- | --- | --- | --- |
| Control | 60 | 18.22±0.49 | - | - |
| 100 μM Res | 60 | 20.29±0.51 | 11.35% | 0.0068 |
| 50 μM BF | 60 | 20.44±0.52 | 12.19% | 0.0045 |
| 250 μM BF | 60 | 20.57±0.53 | 12.93% | 0.0029 |
| 500 μM BF | 60 | 19.90±0.49 | 9.21% | 0.0356 |
